# 8-Thia-1,6-diaza­bicyclo­[4.3.0]nonane-7,9-dione

**DOI:** 10.1107/S1600536811038785

**Published:** 2011-09-30

**Authors:** Bin-bin Xi, Lu Shi, Hai Huang, Qin Wang, Hong-Jun Zhu

**Affiliations:** a Department of Organic Chemistry, College of Science, Nanjing University of Technology, Nanjing 210009, People’s Republic of China; b Department of Applied Chemistry, College of Science, Nanjing University of Technology, Nanjing 210009, People’s Republic of China

## Abstract

There are two independent mol­ecules, *A* and *B*, in the asymmetric unit of the title compound, C_6_H_8_N_2_O_2_S. In the crystal, pairs of inter­molecular S⋯O contacts [3.286 (1) Å] link the *B* mol­ecules into inversion dimers.

## Related literature

For applications of the title compound, see: Yamaguchi *et al.* (1989 ▶). For the synthesis, see: Zhu *et al.* (2011 ▶). For bond-length data, see: Allen *et al.* (1987 ▶). For a review of carbon­yl–carbonyl inter­actions, see: Allen *et al.* (1998 ▶).
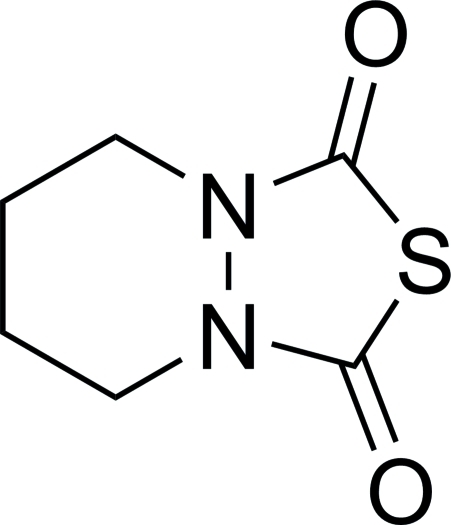


## Experimental

### 

#### Crystal data

C_6_H_8_N_2_O_2_S
*M* *_r_* = 172.20
Triclinic, 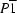

*a* = 7.8400 (16) Å
*b* = 10.464 (2) Å
*c* = 10.514 (2) Å
α = 63.84 (3)°
β = 79.62 (3)°
γ = 89.42 (3)°
*V* = 759.2 (3) Å^3^
*Z* = 4
Mo *K*α radiation
μ = 0.37 mm^−1^
*T* = 293 K
0.30 × 0.20 × 0.10 mm

#### Data collection

Enraf–Nonius CAD-4 diffractometer
Absorption correction: ψ scan (North *et al.*, 1968 ▶) *T*_min_ = 0.896, *T*_max_ = 0.964
3018 measured reflections
2795 independent reflections
2092 reflections with *I* > 2σ(*I*)
*R*_int_ = 0.041
3 standard reflections every 200 reflections  intensity decay: 1%

#### Refinement

*R*[*F* ^2^ > 2σ(*F* ^2^)] = 0.044
*wR*(*F* ^2^) = 0.125
*S* = 1.00
2795 reflections
200 parameters
H-atom parameters constrained
Δρ_max_ = 0.22 e Å^−3^
Δρ_min_ = −0.32 e Å^−3^

### 

Data collection: *CAD-4 EXPRESS* (Enraf–Nonius, 1994 ▶); cell refinement: *CAD-4 EXPRESS*; data reduction: *XCAD4* (Harms & Wocadlo,1995 ▶); program(s) used to solve structure: *SHELXS97* (Sheldrick, 2008 ▶); program(s) used to refine structure: *SHELXL97* (Sheldrick, 2008 ▶); molecular graphics: *SHELXTL* (Sheldrick, 2008 ▶); software used to prepare material for publication: *SHELXTL*.

## Supplementary Material

Crystal structure: contains datablock(s) I, global. DOI: 10.1107/S1600536811038785/lx2196sup1.cif

Structure factors: contains datablock(s) I. DOI: 10.1107/S1600536811038785/lx2196Isup2.hkl

Supplementary material file. DOI: 10.1107/S1600536811038785/lx2196Isup3.cml

Additional supplementary materials:  crystallographic information; 3D view; checkCIF report

## Figures and Tables

**Fig. 1.**
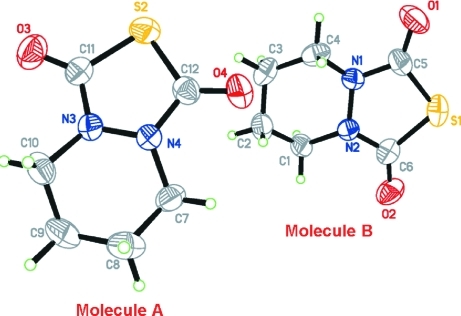
The molecular structure of the title compound with the atom numbering scheme. Displacement ellipsoids are drawn at the 50% probability level. H atoms are presented as small spheres of arbitrary radius.

**Fig. 2.**
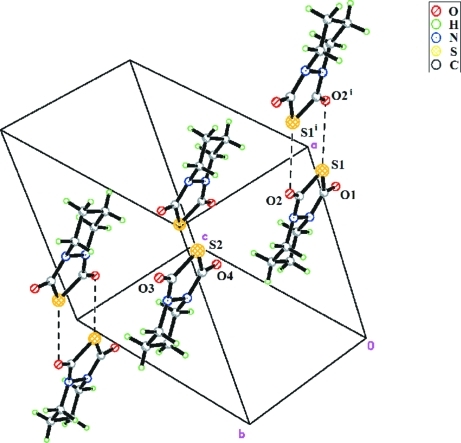
A view of the S···O interactions (dotted lines) in the crystal structure of the title compound. [Symmetry codes: (i) - x, - y + 2, - z.]

## supplementary crystallographic information

### Comment

The title compound, 8-thia-1,6-diazabicyclo[4.3.0]nonane-7,9-dione, is an important intermediate for a kind of manufacturing agrochemical, especially for Fluthiacet-methyl as a super-effective, wide-spectral and safe herbicide (Yamaguchi *et al.*, 1989). We report herein the crystal structure of the title compound.

The asymmetric unit of the title compound is shown in Fig. 1 and there are two independent unique molecules [labeled A & B]. The bond lengths and angles are within normal ranges (Allen *et al.*, 1987). The crystal packing (Fig. 2) is stabilized by an intermolecular S···O interaction between the sulfur and the O atom of the carbonyl group interpreted as similar to type-II carbonyl–carbonyl interaction (Allen *et al.*, 1998), with S1···O2^i^ distance of 3.286 (1) Å.

### Experimental

The title compound was prepared  according to reported in literature (Zhu *et al.*, 2011). Single crystals suitable for X-ray diffraction were prepared by slow evaporation of a solution of the title compound in ethanol at room temperature for ca. 6 d.

### Refinement

All H atoms were positioned geometrically and refined using a riding model, with C—H = 0.95 Å and *U*_iso_(H) =1.5*U*_eq_(C).

### Crystal data

**Table:**

C_6_H_8_N_2_O_2_S	*Z* = 4
*M**_r_* = 172.20	*F*(000) = 360
Triclinic, *P*1	*D*_x_ = 1.507 Mg m^−^^3^
Hall symbol: -P 1	Mo *K*α radiation, λ = 0.71073 Å
*a* = 7.8400 (16) Å	Cell parameters from 25 reflections
*b* = 10.464 (2) Å	θ = 10–13°
*c* = 10.514 (2) Å	µ = 0.37 mm^−^^1^
α = 63.84 (3)°	*T* = 293 K
β = 79.62 (3)°	Block, colourless
γ = 89.42 (3)°	0.30 × 0.20 × 0.10 mm
*V* = 759.2 (3) Å^3^	

### Data collection

**Table:**

Enraf–Nonius CAD-4 diffractometer	2092 reflections with *I* > 2σ(*I*)
Radiation source: fine-focus sealed tube	*R*_int_ = 0.041
graphite	θ_max_ = 25.4°, θ_min_ = 2.2°
ω/2θ scans	*h* = 0→9
Absorption correction: ψ scan (North *et al.*, 1968)	*k* = −12→12
*T*_min_ = 0.896, *T*_max_ = 0.964	*l* = −12→12
3018 measured reflections	3 standard reflections every 200 reflections
2795 independent reflections	intensity decay: 1%

### Refinement

**Table:**

Refinement on *F*^2^	Secondary atom site location: difference Fourier map
Least-squares matrix: full	Hydrogen site location: difference Fourier map
*R*[*F*^2^ > 2σ(*F*^2^)] = 0.044	H-atom parameters constrained
*wR*(*F*^2^) = 0.125	*w* = 1/[σ^2^(*F*_o_^2^) + (0.077*P*)^2^] where *P* = (*F*_o_^2^ + 2*F*_c_^2^)/3
*S* = 1.00	(Δ/σ)_max_ < 0.001
2795 reflections	Δρ_max_ = 0.22 e Å^−^^3^
200 parameters	Δρ_min_ = −0.32 e Å^−^^3^
0 restraints	Extinction correction: *SHELXL*, Fc^*^=kFc[1+0.001xFc^2^λ^3^/sin(2θ)]^-1/4^
Primary atom site location: structure-invariant direct methods	Extinction coefficient: 0.327 (16)

### Special details

**Table:**

Geometry. All e.s.d.'s (except the e.s.d. in the dihedral angle between two l.s. planes) are estimated using the full covariance matrix. The cell e.s.d.'s are taken into account individually in the estimation of e.s.d.'s in distances, angles and torsion angles; correlations between e.s.d.'s in cell parameters are only used when they are defined by crystal symmetry. An approximate (isotropic) treatment of cell e.s.d.'s is used for estimating e.s.d.'s involving l.s. planes.
Refinement. Refinement of *F*^2^ against ALL reflections. The weighted *R*-factor *wR* and goodness of fit *S* are based on *F*^2^, conventional *R*-factors *R* are based on *F*, with *F* set to zero for negative *F*^2^. The threshold expression of *F*^2^ > σ(*F*^2^) is used only for calculating *R*-factors(gt) *etc*. and is not relevant to the choice of reflections for refinement. *R*-factors based on *F*^2^ are statistically about twice as large as those based on *F*, and *R*- factors based on ALL data will be even larger.

### Fractional atomic coordinates and isotropic or equivalent isotropic displacement parameters (Å^2^)

**Table:**

	*x*	*y*	*z*	*U*_iso_*/*U*_eq_	
S1	−0.09731 (11)	0.82576 (8)	0.04086 (8)	0.0598 (3)	
O1	−0.3185 (3)	0.5944 (3)	0.1791 (3)	0.0737 (7)	
O2	0.2479 (3)	0.8734 (2)	−0.0219 (2)	0.0632 (6)	
N1	−0.0292 (2)	0.5625 (2)	0.1507 (2)	0.0416 (5)	
N2	0.1318 (3)	0.6432 (2)	0.1083 (2)	0.0411 (5)	
C1	0.2837 (3)	0.5721 (3)	0.0768 (3)	0.0464 (6)	
H1A	0.3892	0.6300	0.0591	0.056*	
H1B	0.2803	0.5604	−0.0092	0.056*	
C2	0.2842 (4)	0.4278 (3)	0.2034 (3)	0.0547 (7)	
H2A	0.3027	0.4404	0.2860	0.066*	
H2B	0.3789	0.3771	0.1791	0.066*	
C3	0.1134 (4)	0.3408 (3)	0.2418 (3)	0.0529 (7)	
H3A	0.1002	0.3205	0.1623	0.063*	
H3B	0.1137	0.2505	0.3261	0.063*	
C4	−0.0370 (4)	0.4199 (3)	0.2726 (3)	0.0491 (7)	
H4A	−0.1456	0.3669	0.2878	0.059*	
H4B	−0.0326	0.4290	0.3599	0.059*	
C5	−0.1691 (3)	0.6421 (3)	0.1356 (3)	0.0497 (7)	
C6	0.1243 (4)	0.7859 (3)	0.0365 (3)	0.0462 (6)	
S2	0.16047 (9)	0.00770 (8)	0.62686 (8)	0.0517 (3)	
O3	0.3930 (3)	−0.1856 (2)	0.6988 (2)	0.0625 (6)	
O4	0.1494 (2)	0.2796 (2)	0.5788 (2)	0.0585 (5)	
N3	0.4698 (2)	0.0396 (2)	0.6615 (2)	0.0396 (5)	
N4	0.3934 (3)	0.1675 (2)	0.6431 (2)	0.0379 (5)	
C7	0.5149 (3)	0.2948 (3)	0.5815 (3)	0.0462 (6)	
H7A	0.4534	0.3749	0.5837	0.055*	
H7B	0.5648	0.3187	0.4817	0.055*	
C8	0.6568 (4)	0.2672 (3)	0.6669 (3)	0.0567 (8)	
H8A	0.6083	0.2567	0.7629	0.068*	
H8B	0.7430	0.3483	0.6203	0.068*	
C9	0.7431 (3)	0.1341 (3)	0.6786 (3)	0.0541 (7)	
H9A	0.8298	0.1158	0.7384	0.065*	
H9B	0.8015	0.1481	0.5833	0.065*	
C10	0.6102 (3)	0.0070 (3)	0.7438 (3)	0.0502 (7)	
H10A	0.6655	−0.0756	0.7418	0.060*	
H10B	0.5628	−0.0153	0.8437	0.060*	
C11	0.3614 (3)	−0.0634 (3)	0.6686 (3)	0.0433 (6)	
C12	0.2314 (3)	0.1752 (3)	0.6121 (3)	0.0412 (6)	

### Atomic displacement parameters (Å^2^)

**Table:**

	*U*^11^	*U*^22^	*U*^33^	*U*^12^	*U*^13^	*U*^23^
S1	0.0698 (5)	0.0513 (5)	0.0626 (5)	0.0262 (4)	−0.0203 (4)	−0.0268 (4)
O1	0.0382 (12)	0.0922 (17)	0.0976 (17)	0.0078 (11)	−0.0077 (11)	−0.0508 (14)
O2	0.0749 (14)	0.0403 (11)	0.0593 (13)	−0.0102 (10)	0.0012 (11)	−0.0138 (10)
N1	0.0357 (11)	0.0393 (11)	0.0448 (12)	0.0011 (9)	−0.0052 (9)	−0.0153 (10)
N2	0.0369 (11)	0.0349 (11)	0.0453 (12)	0.0006 (9)	−0.0038 (9)	−0.0139 (9)
C1	0.0375 (14)	0.0472 (15)	0.0502 (15)	0.0045 (11)	0.0008 (11)	−0.0213 (13)
C2	0.0508 (17)	0.0486 (16)	0.0617 (18)	0.0155 (13)	−0.0147 (14)	−0.0209 (14)
C3	0.073 (2)	0.0344 (14)	0.0467 (15)	0.0053 (13)	−0.0151 (14)	−0.0130 (12)
C4	0.0545 (16)	0.0431 (15)	0.0406 (14)	−0.0101 (12)	−0.0044 (12)	−0.0120 (12)
C5	0.0418 (16)	0.0638 (18)	0.0531 (16)	0.0122 (13)	−0.0109 (13)	−0.0343 (15)
C6	0.0585 (17)	0.0418 (15)	0.0362 (13)	0.0039 (13)	−0.0048 (12)	−0.0172 (12)
S2	0.0443 (4)	0.0511 (4)	0.0589 (5)	−0.0031 (3)	−0.0175 (3)	−0.0208 (3)
O3	0.0769 (15)	0.0407 (11)	0.0712 (14)	0.0081 (10)	−0.0188 (11)	−0.0247 (10)
O4	0.0494 (11)	0.0536 (12)	0.0682 (13)	0.0176 (10)	−0.0212 (10)	−0.0200 (10)
N3	0.0358 (11)	0.0354 (11)	0.0451 (12)	0.0058 (9)	−0.0080 (9)	−0.0159 (9)
N4	0.0328 (10)	0.0335 (11)	0.0445 (12)	0.0028 (8)	−0.0076 (9)	−0.0150 (9)
C7	0.0446 (15)	0.0356 (13)	0.0543 (16)	−0.0040 (11)	−0.0011 (12)	−0.0195 (12)
C8	0.0418 (15)	0.0667 (19)	0.0708 (19)	−0.0067 (14)	−0.0050 (14)	−0.0409 (16)
C9	0.0312 (13)	0.076 (2)	0.0632 (18)	0.0066 (13)	−0.0108 (13)	−0.0377 (16)
C10	0.0381 (14)	0.0576 (16)	0.0520 (16)	0.0119 (12)	−0.0132 (12)	−0.0204 (14)
C11	0.0467 (15)	0.0393 (15)	0.0413 (14)	0.0016 (12)	−0.0056 (11)	−0.0167 (11)
C12	0.0386 (13)	0.0453 (14)	0.0359 (13)	0.0049 (12)	−0.0095 (11)	−0.0138 (11)

### Geometric parameters (Å, °)

**Table:**

S1—C5	1.771 (3)	S2—C12	1.776 (3)
S1—C6	1.780 (3)	S2—C11	1.779 (3)
O1—C5	1.203 (3)	O3—C11	1.208 (3)
O2—C6	1.209 (3)	O4—C12	1.208 (3)
N1—C5	1.360 (3)	N3—C11	1.347 (3)
N1—N2	1.411 (3)	N3—N4	1.409 (3)
N1—C4	1.470 (3)	N3—C10	1.467 (3)
N2—C6	1.351 (3)	N4—C12	1.360 (3)
N2—C1	1.463 (3)	N4—C7	1.464 (3)
C1—C2	1.510 (4)	C7—C8	1.501 (4)
C1—H1A	0.9700	C7—H7A	0.9700
C1—H1B	0.9700	C7—H7B	0.9700
C2—C3	1.514 (4)	C8—C9	1.508 (4)
C2—H2A	0.9700	C8—H8A	0.9700
C2—H2B	0.9700	C8—H8B	0.9700
C3—C4	1.503 (4)	C9—C10	1.514 (4)
C3—H3A	0.9700	C9—H9A	0.9700
C3—H3B	0.9700	C9—H9B	0.9700
C4—H4A	0.9700	C10—H10A	0.9700
C4—H4B	0.9700	C10—H10B	0.9700
			
C5—S1—C6	91.50 (13)	C12—S2—C11	91.50 (12)
C5—N1—N2	114.0 (2)	C11—N3—N4	114.76 (19)
C5—N1—C4	121.3 (2)	C11—N3—C10	122.2 (2)
N2—N1—C4	114.5 (2)	N4—N3—C10	114.6 (2)
C6—N2—N1	114.8 (2)	C12—N4—N3	114.5 (2)
C6—N2—C1	121.8 (2)	C12—N4—C7	121.1 (2)
N1—N2—C1	115.11 (19)	N3—N4—C7	115.35 (19)
N2—C1—C2	109.5 (2)	N4—C7—C8	109.7 (2)
N2—C1—H1A	109.8	N4—C7—H7A	109.7
C2—C1—H1A	109.8	C8—C7—H7A	109.7
N2—C1—H1B	109.8	N4—C7—H7B	109.7
C2—C1—H1B	109.8	C8—C7—H7B	109.7
H1A—C1—H1B	108.2	H7A—C7—H7B	108.2
C1—C2—C3	110.6 (2)	C7—C8—C9	111.1 (2)
C1—C2—H2A	109.5	C7—C8—H8A	109.4
C3—C2—H2A	109.5	C9—C8—H8A	109.4
C1—C2—H2B	109.5	C7—C8—H8B	109.4
C3—C2—H2B	109.5	C9—C8—H8B	109.4
H2A—C2—H2B	108.1	H8A—C8—H8B	108.0
C4—C3—C2	110.8 (2)	C8—C9—C10	110.7 (2)
C4—C3—H3A	109.5	C8—C9—H9A	109.5
C2—C3—H3A	109.5	C10—C9—H9A	109.5
C4—C3—H3B	109.5	C8—C9—H9B	109.5
C2—C3—H3B	109.5	C10—C9—H9B	109.5
H3A—C3—H3B	108.1	H9A—C9—H9B	108.1
N1—C4—C3	109.9 (2)	N3—C10—C9	109.7 (2)
N1—C4—H4A	109.7	N3—C10—H10A	109.7
C3—C4—H4A	109.7	C9—C10—H10A	109.7
N1—C4—H4B	109.7	N3—C10—H10B	109.7
C3—C4—H4B	109.7	C9—C10—H10B	109.7
H4A—C4—H4B	108.2	H10A—C10—H10B	108.2
O1—C5—N1	125.0 (3)	O3—C11—N3	126.2 (3)
O1—C5—S1	125.5 (2)	O3—C11—S2	124.5 (2)
N1—C5—S1	109.5 (2)	N3—C11—S2	109.34 (18)
O2—C6—N2	125.7 (3)	O4—C12—N4	125.5 (2)
O2—C6—S1	125.1 (2)	O4—C12—S2	125.4 (2)
N2—C6—S1	109.2 (2)	N4—C12—S2	109.10 (18)
			
C5—N1—N2—C6	−11.3 (3)	C11—N3—N4—C12	−10.1 (3)
C4—N1—N2—C6	−157.2 (2)	C10—N3—N4—C12	−159.0 (2)
C5—N1—N2—C1	−160.4 (2)	C11—N3—N4—C7	−157.8 (2)
C4—N1—N2—C1	53.6 (3)	C10—N3—N4—C7	53.3 (3)
C6—N2—C1—C2	160.1 (2)	C12—N4—C7—C8	162.2 (2)
N1—N2—C1—C2	−53.2 (3)	N3—N4—C7—C8	−52.4 (3)
N2—C1—C2—C3	54.1 (3)	N4—C7—C8—C9	53.6 (3)
C1—C2—C3—C4	−56.3 (3)	C7—C8—C9—C10	−56.1 (3)
C5—N1—C4—C3	164.3 (2)	C11—N3—C10—C9	161.2 (2)
N2—N1—C4—C3	−52.4 (3)	N4—N3—C10—C9	−52.4 (3)
C2—C3—C4—N1	53.8 (3)	C8—C9—C10—N3	53.8 (3)
N2—N1—C5—O1	−171.7 (3)	N4—N3—C11—O3	−171.8 (2)
C4—N1—C5—O1	−28.3 (4)	C10—N3—C11—O3	−25.6 (4)
N2—N1—C5—S1	8.8 (3)	N4—N3—C11—S2	7.9 (3)
C4—N1—C5—S1	152.26 (19)	C10—N3—C11—S2	154.20 (19)
C6—S1—C5—O1	176.8 (3)	C12—S2—C11—O3	176.4 (2)
C6—S1—C5—N1	−3.76 (19)	C12—S2—C11—N3	−3.33 (19)
N1—N2—C6—O2	−172.2 (2)	N3—N4—C12—O4	−172.5 (2)
C1—N2—C6—O2	−25.3 (4)	C7—N4—C12—O4	−26.9 (4)
N1—N2—C6—S1	7.8 (3)	N3—N4—C12—S2	6.9 (3)
C1—N2—C6—S1	154.63 (19)	C7—N4—C12—S2	152.55 (18)
C5—S1—C6—O2	177.7 (3)	C11—S2—C12—O4	177.4 (2)
C5—S1—C6—N2	−2.26 (19)	C11—S2—C12—N4	−2.03 (19)
